# Optical Assessment of Nociceptive TRP Channel Function at the Peripheral Nerve Terminal

**DOI:** 10.3390/ijms22020481

**Published:** 2021-01-06

**Authors:** Fernando Aleixandre-Carrera, Nurit Engelmayer, David Ares-Suárez, María del Carmen Acosta, Carlos Belmonte, Juana Gallar, Víctor Meseguer, Alexander M. Binshtok

**Affiliations:** 1 Instituto de Neurociencias, Universidad Miguel Hernández—CSIC, 03550 San Juan de Alicante, Spain; f.aleixandre@umh.es (F.A.-C.); dares@umh.es (D.A.-S.); mcarmen.acosta@umh.es (M.d.C.A.); carlos.belmonte@umh.es (C.B.); juana.gallar@umh.es (J.G.); 2 Department of Medical Neurobiology, Institute for Medical Research Israel—Canada, Hadassah School of Medicine, The Hebrew University, Jerusalem 91123, Israel; nurit.engelmayer@mail.huji.ac.il (N.E.); alexander.binshtok@mail.huji.ac.il (A.M.B.); 3 The Edmond and Lily Safra Center for Brain Sciences, The Hebrew University of Jerusalem, Jerusalem 9190401, Israel; 4 Instituto de Investigación Sanitaria y Biomédica de Alicante, 03550 San Juan de Alicante, Spain

**Keywords:** peripheral nerve terminals, TRP channels, nociception, pain, optical recording, opto-pharmacology

## Abstract

Free nerve endings are key structures in sensory transduction of noxious stimuli. In spite of this, little is known about their functional organization. Transient receptor potential (TRP) channels have emerged as key molecular identities in the sensory transduction of pain-producing stimuli, yet the vast majority of our knowledge about sensory TRP channel function is limited to data obtained from in vitro models which do not necessarily reflect physiological conditions. In recent years, the development of novel optical methods such as genetically encoded calcium indicators and photo-modulation of ion channel activity by pharmacological tools has provided an invaluable opportunity to directly assess nociceptive TRP channel function at the nerve terminal.

## 1. Introduction

**Figure 1:**
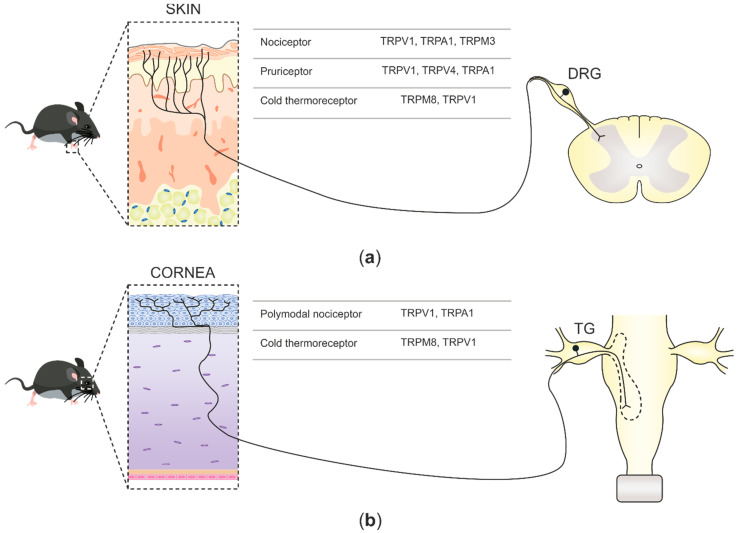
Schematic illustration depicting somatosensory innervation and their projections to the central nervous system (CNS). (**a**) Somatosensory nerve endings innervating the skin project to the dorsal horn second-order neurons in the spinal cord. Three functional types of sensory neurons innervating the skin are represented along with the transient receptor potential (TRP) channels expressed. (**b**) Corneal somatosensory nerve endings densely populate the corneal epithelium, reach the trigeminal ganglia (TG) through the ophthalmic nerve, and project, together with other primary nociceptive neurons innervating the face, to second-order CNS neurons at the spinal part of the trigeminal nuclei in the brainstem. Two functional forms of corneal sensory neurons and the TRP channels that they express are represented.

In mammals, environmental information is predominantly relayed by peripheral neurons of the somatosensory system. Such neurons are cellular sensors, transforming mechanical, thermal, and chemical stimuli into electrical signals that progress to the central nervous system (CNS) as action potentials [1]. Sensory neurons in mammals are pseudo-unipolar, possessing a single axon with two distinct branches: the peripheral branch which extends and innervates the target organs such as skin, viscera, and mucosae, and the central branch which relays the collected information to second-order neurons of the CNS in the spinal dorsal horn or brainstem sensory nuclei [2]. The cell bodies of primary sensory neurons innervate the body group together in segmental order in dorsal root ganglia (DRGs), which are found lateral to the spinal cord (Figure 1a). Primary sensory neuron cell bodies, which innervate the head and face, cluster in the trigeminal ganglia (TG) juxtaposed to the brain [3] (Figure 1b). 

Traditionally, sensory nerve fibers have been classified into four types according to their conduction velocities [4]. Aα- and Aβ-fibers show highly myelinated axons and fast conduction velocities, Aδ are mildly myelinated and have medium conduction velocities, whereas C-fibers lack myelin and have slow conduction velocities [5]. The sensory neurons responsible for detecting potentially harmful stimuli mostly belong to the C-fiber or Aδ class and are known as nociceptors [6]. Aδ-nociceptors underlie the rapid component of pain immediately upon tissue damage [4], while C-nociceptors contribute to later stages of chronic pain development, neurogenic inflammation, and sensitization [7].

The ability of nociceptors to behave as noxious stimuli detectors relies on the presence of specialized transducing molecules at their peripheral nerve terminals capable of transforming the harmful physical (thermal and mechanical) and chemical stimuli into generator potentials [4,8]. Upon nerve terminal stimulation, the output signal conveying to the CNS depends on the properties of transducer channels such as transient receptor potentials (TRPs), acid-sensing ion channels (ASICs), Piezo, and TACAN [9,10,11,12,13,14,15], which produce generator potentials. Voltage-gated channels subsequently translate it into action potential firing. TRP ion channels are among the most studied transducer channels expressed in nociceptors and play a pivotal role in the study of pain [4].

To date, the main component of our knowledge on transducer TRP channel function at the nerve terminal has been inferred from experimental data obtained from in vitro transduction models lacking physiological conditions such as cell lines heterologously expressing TRP channels or cultured primary sensory neurons [16]. Although this provided invaluable information on the roles TRP channels play in nociception, the possible alteration of expression, distribution, and modulation by endogenous factors of TRP channels under non-physiological conditions [17,18], and other factors such as distinct morphology between the nerve terminal and soma, might account for different coding of noxious stimuli by TRP channels in their physiological environment. Indeed, a recently published computational model revealed that the structure of peripheral nociceptive terminal tree determines the output of the nociceptive neurons at the central terminal [19]. Since in vitro models do not recapitulate the entire nerve terminal complexity, there is an obvious need to assess TRP channel functionality directly on the nerve terminal in more physiological conditions.

Until recent years, the most direct experimental approaches in the study of TRP channels’ functionality at the nerve terminal were based on extracellular electrophysiological recordings in conjunction with the application of TRP channels’ agonists and antagonists on skin-nerve or corneal nerve preparations [20,21,22,23,24]. While these experimental approaches provide a high temporal resolution on peripheral nerve activity, they do not allow for enough spatial resolution regarding where at the nerve terminal this activity occurs. In this respect, the recent development of optical recording techniques is a big step in overcoming these significant technical limitations [25,26,27,28,29,30].

Additionally, attempting to study the functionality of nociceptive TRP channels in vivo requires prolonged pharmacological or genetically mediated changes in channel function [31,32]. However, ion channel kinetics are very fast, thus these methods might not reflect the full effect of ion channels on cell physiology. Thus, assessing ion channel activity by experimental approaches should be performed with a high temporal resolution, which necessitates new approaches for selective and fast ways of modulating channel activity in the live animal. In this regard, the development of optogenetics, nanoparticle-based methods, and opto-pharmacological tools undoubtedly provides new routes for studying the contribution of ion channels to cell physiology in vivo and establishing new means to precisely control neuronal activity [33,34,35,36,37]. Moreover, these methods have multiple uses in experimental pain neurobiology, turning out advantageous over traditional methods in clinical applications. For instance, neural stimulation for the treatment of pain is invasive and electrodes are often difficult to reposition [38]; likewise, chemical stimulation is difficult to constrain to individual cells and therefore, has limited spatial and temporal accuracy. On the contrary, direct modulation of ion channels with light allows for controlled stimulation with unparalleled spatial and temporal precision [39].

This article provides an overview of the latest optical tools applied in optical control and optical recording of nociceptive TRP channel activity at the nerve terminal.

## 2. Nociceptive TRP Channels at Sensory Nerve Fibers

Several members of the TRP family have been identified as primary molecular transducers on nociceptive nerve fibers. Based on their sequence homology, the mammalian TRP superfamily is comprised of six subfamilies with 28 cation-permeable channels organized as canonical (TRPC), vanilloid (TRPV), ankyrin (TRPA), melastatin (TRPM), polycystin (TRPP), and mucolipin (TRPML) [40]. Overall, this TRP superfamily of channels is involved in multiple physiological and pathophysiological processes, however, based on their ability to transduce specific physical and chemical noxious stimuli, only a few members belonging to TRPV, TRPA, and TRPM families have been classified as nociceptive TRPs [41].

TRPV1 is the first identified and most well-characterized as the molecular entity underlying the detection of capsaicin in sensory neurons, the pungent ingredient in hot chili [42]. Besides capsaicin, TRPV1 can be activated by a large number of physical and chemical stimuli such as noxious temperature (>42 °C), pungent plant products, low pH, and animal toxins. Moreover, TRPV1 possesses the ability to integrate distinct noxious stimuli at the protein level, archetypal of a polymodal receptor channel [43]. In the peripheral nervous system, TRPV1 is mainly expressed in small- and medium-diameter nociceptive neurons from DRG and TG, such as peptidergic and non-peptidergic C-fibers, as well as in Aδ-fibers [44,45].

TRPA1 is the sole mammalian member of the TRPA subfamily associated with pain transduction and, similarly to TRPV1, behaves as a polymodal receptor by integrating, at the molecular level, the transduction of distinct types of noxious stimuli. It is expressed in small-diameter DRG and TG sensory neurons that, in addition, co-express characteristic nociceptive markers such as TRPV1, substance P, and/or calcitonin gene-related peptide (CGRP) [46]. These features, along with activation by temperatures below 18 °C, suggest that TRPA1 acts as a noxious-cold detector in nociceptive sensory neurons [47,48]. Some studies report prominent noxious-cold-sensing impairments of TRPA1-knockout mice [49], whereas others do not find a distinct phenotype in cold-sensing behaviors [50,51]. Recent evidence points toward the role of TRPA1 in sensing acute noxious heat [52,53]. TRPA1 can also be activated by a plethora of irritants and injurious compounds, including allyl isothiocyanate found in mustard oil and wasabi [47,54], nicotine [55], bacterial endotoxins [56], or reactive oxygen species (ROS) [57,58,59], which are fundamental for tissue damage and cellular stress signaling. The vast spectrum of potentially harmful stimuli that activate TRPA1 has led to the notion that TRPA1 acts as a molecular sensor for cellular stress and tissue damage [60].

TRPM3 is a noxious heat-activated channel expressed in a large subset of DRG and TG sensory neurons along with nociceptors [61]. Its activation threshold is slightly shifted towards higher temperatures in comparison to TRPV1 and can also be activated by the neurosteroid pregnenolone sulfate or the more effective synthetic agonist, CIM0216 [62]. Mice lacking TRPM3 channels display an impairment in evasion behaviors to noxious heat as well as in the development of heat hyperalgesia [61]. Likewise, pharmacological blockade of TRPM3 produces a deficit in noxious heat sensitivity in mice [63]. Interestingly, ablation of TRPV1 and TRPM3 genes does not completely suppress the avoidance behavior to noxious heat, indicating that additional detection mechanisms must exist. Only when TRPV1, TRPM3, and TRPA1 are deleted do mice completely lack acute noxious heat responses to avoid burn injury in the tail-immersion assay [52]. Recent work has revealed that TRPM2 underlies residual heat responses (above 45 °C) in dissociated primary sensory neurons. Its role in noxious heat-sensing in vivo needs yet to be elucidated [64,65,66].

TRPM8 was the first characterized thermo-TRP channel activated by cold, as its conductance sharply increases as temperature drops [67,68]. Simultaneously, it was found to be activated by natural compounds known for evoking "fresh" sensations, such as eucalyptol, menthol [68], and the more potent agonist icilin. TRPM8 is expressed in a subpopulation of Aδ- and C-fibers emanating from DRG and TG small-diameter sensory neurons [67]. Several functional assays established that around 20% of DRG TRPM8-expressing neurons also responded to the TRPV1 agonist capsaicin despite not demonstrating expression of CGRP or substance P, known markers for nociceptor identity. Strikingly, this proportion is raised to 45% in TG TRPM8-positive neurons innervating the cornea, while the majority of them also expressed substance P [69]. Shortly after the first characterizations of TRPM8-null mice, it was solidly understood that the TRPM8 ion channel is a central protagonist in mild cold thermosensation [70,71,72].

In addition to transducer channels that underlie the receptor potential generation, sensory nerve fibers express a large variety of voltage- and ligand-gated ion channels, primarily voltage-gated sodium channels (Navs), voltage-gated potassium channels (Kvs), voltage-gated calcium channels (VGCCs), and hyperpolarization-activated cyclic nucleotide–gated (HCN) channels. These ion channels define nerve terminal excitability by mediating the formation of the nerve impulses that are conveyed to second-order sensory neurons at the CNS [73].

### 2.1. TRP Channels at Skin Nerve Endings

Sensory neurons innervating the skin can be categorized into different groups based on their functional profiles [74] (Figure 1a): nociceptors, pruriceptors, thermoreceptors, and low-threshold mechanoreceptors (LTMRs). Nociceptors detect high-intensity stimuli such as mechanical forces, harmful heating, and potentially noxious chemicals. The ability of the nociceptive neurons to be activated by a wide variety of stimuli is largely due to the expression of TRPV1, TRPA1, and TRPM3 [42,61,75]. Pruriceptors, on the other hand, form a subset of DRG neurons [76] in which the presence of functional TRPV1, TRPA1, and TRPV4 are essential for initiating itch sensation [77,78,79]. Thermoreceptors encode temperature changes, being TRPM8, the initial cold-activated TRP channel characterized [67,68], expressed in a subset of Aδ- and C-fibers. These cold thermoreceptors activate upon cooling in the innocuous range (15–28 °C) and constitute the cellular substrate for cold detection in mammals as the ablation of TRPM8-expressing neurons leads to a robust impairment in cold-avoidance behavior [80,81]. The neurons activated by non-noxious warmth were proposed to express TRPV3 and TRPV4, as in heterologous expression systems, these channels show activation thresholds of 25 and 35 °C, respectively [82,83,84,85]. Nonetheless, the extremely low expression of TRPV3 and TRPV4 in sensory neurons advise against their role in thermosensation. Recent evidence points towards the inhibition of TRPM8-expressing cold thermoreceptors as an indispensable mechanism for warmth perception in mice [22]. Lastly, LTMRs found in hairy and glabrous skin are profusely diverse. These receptors also respond to weak mechanical force, and detect and encode innocuous mechanical stimuli (touch) without TRP ion channels, contributing to mechanotransduction [75,86].

### 2.2. TRP Channels at Corneal Nerve Terminals

Corneal innervation is derived from sensory trigeminal neurons that have been categorized into three functional types on the basis of their ability to detect distinct physical and chemical stimuli [87]. Aside from differences in the expression of a variety of ion channels and other proteins, these neurons differ specifically in the expression of TRP channels at the cell body, axonal branches, and nerve terminals. Extracellular recordings of skin and corneal nerve terminals have significantly contributed to our current knowledge of nociceptive TRP channels’ functions at the nerve terminal and subsequently, sensory neurons innervating the cornea have been classified into three main functional types [73] (Figure 1b): (1) polymodal nociceptors, the most abundant population (70%), which respond to a wide spectrum of thermal, chemical, and mechanical noxious stimuli and express TRPV1 and TRPA1 channels. (2) Mechano-nociceptor subpopulation of corneal nociceptor neurons (10–15%), that respond only to mechanical forces, express the Piezo2 ion channel as the main transducer receptor [13,88]. (3) Cold thermoreceptors (10–15%) are characterized by the expression of the cold-sensing TRPM8 channel, which is activated by small temperature drops in addition to moderate osmolarity increases [23,89]. Interestingly, both stimuli are naturally activated by tear evaporation in dry external environments, leading to the activation of cold thermoreceptors and subsequently, the propagated action potentials are used by higher-order brainstem neurons to reflexively regulate tear flow and blinking rate [23,89,90,91]. Furthermore, corneal cold thermoreceptors can be divided into two different subclasses [92,93,94]. Low-threshold cold thermoreceptors (LTC) highly express TRPM8 and display continuous nerve impulse firing at normal corneal temperatures (34–35 °C), which increases under small temperature drops (≤0.5 °C). The other functional group consists of high-threshold cold thermoreceptors (HTC) that remain silent at corneal temperatures above 29 °C and require stronger cooling stimulation than LTC neurons in order to be activated. Importantly, HTC corneal nerve fibers display a smaller expression of TRPM8 than LTC neurons and possibly contain TRPV1 channels [93,94]. Indeed, a high proportion of TRPM8-expressing corneal nerve fibers also express the nociceptive TRPV1 channel [69].

## 3. Opto-Pharmacological Control of Nociceptive TRP Channel Activity at the Nerve Terminal

Conventional pharmacological strategies for the research of ion channels are limited by low selectivity and insufficient control over the concentration at their targets [95]. With the aim of overcoming these hurdles, a plethora of photo-sensitive drugs with the ability to change their structure upon irradiation with light have been developed in recent years [34,96]. Generally, these opto-pharmacological agents constitute a novel technique known as opto-pharmacology or photo-pharmacology, which permits non-invasive, precise spatiotemporal optical control of neural activity [34,97,98]. Opto-pharmacological tools have been generated employing three main strategies, caged ligands, photochromic ligands, and in combination with genetic tools, photochromic tethered ligands (PTLs) [99]. Here, we provide a brief overview of these strategies. For a further detailed discussion, the reader is referred to other recent reviews [33,39].

Caged ligands are composed of receptor agonist or antagonist attached to a light-sensitive group, which prevents ligands from their interaction with their receptors and thus they remain biologically inactive in darkness, while light irradiation induces uncaging of the molecule by means of photolysis, which releases the ligand rapidly and locally with high spatial and temporal precision [34]. Caged ligands have been widely used in neuroscience, mainly in the photo-control of neurotransmitter receptors, as well as neuropeptide receptors [100,101,102,103,104]. Notably, caged ligands show high stability before photolysis and the rate of uncaging is faster than the kinetics of the targeted biological processes. Nevertheless, photolysis is a single-shot process that lacks reversibility, thus not allowing for cyclic signal activation.

Photochromic ligands (chemical photo-switches) are typically generated on the basis of a receptor-ligand attached to a photo-isomerizable group. Photo-isomerization between *cis-* and *trans*-states of the photochromic moiety is achieved by irradiation of light at different wavelengths, which in turn allows for reversible on–off optical control of protein targets, either a channel or a receptor. Of note, although more gradually, a conformational transition from *cis*- to the thermodynamically more stable *trans*-state occurs in the absence of light as well [99]. Azobenzene is the most widely used synthetic photo-switch due to relevant features such as fast isomerization (picoseconds range), light absorption at low intensities, and an advantage over caged ligands is reversibility of photo-switching. However, in a similar way to caged ligands and conventional drugs, photochromic ligands lack specificity for a given molecular target. Overall, azobenzene-containing ligands have been successfully assayed for precise spatiotemporal optical control of Navs and Kvs as well as TRP channels [39,96,105,106].

PTLs overcome the limitation of target specificity by the attachment of a synthetic photo-sensitive ligand onto a genetically modified protein to allow photo-modulation of only specific channels or receptors. This approach relies on incorporation into the receptor of cysteines in the close surroundings of the ligand-binding site, and additionally, requires the presence of a maleimide group into the photo-switchable ligand for this to couple to the cysteine residue. In this manner, azobenzene-containing PTLs have been particularly useful as photo-modulators of a number of neural receptors and ion channels [34,107,108,109]. Nevertheless, a more extended use of this strategy may be limited by the technically demanding requirement of two exogenous components, the photo-switchable ligand and gene encoding the target receptor [34].

**Figure 2:**
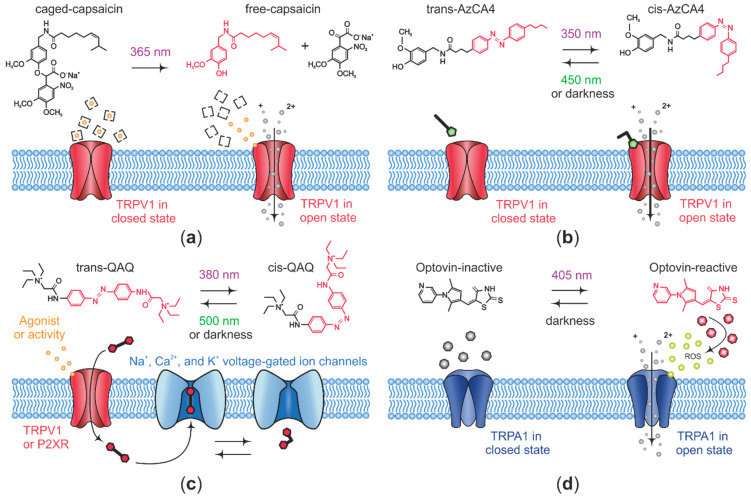
Illustration showing several strategies employed to assess nociceptive transient receptor potential (TRP) channel function using opto-chemicals. (**a**) Caged capsaicin remains inactive until photolysis with 365 nm light releases capsaicin, thus activating the transient receptor potential vanilloid receptor 1 (TRPV1) channels by a non-reversible process. (**b**) AzCA4 (Azo-capsaicin 4) is a photo-isomerizable TRPV1 agonist that activates TRPV1 channels at its *cis* configuration upon ultraviolet light illumination, whereas it remains inactive after photo-isomerization with blue light or in the dark. (**c**) QAQ (Quaternary ammonium-azobenzene-quaternary ammonium) is a photo-isomerizable compound that permeates open TRPV1 and ATP-gated purinergic receptor 2X (P2XR) channels, and selectively accumulates inside the peripheral nociceptors where it blocks voltage-gated sodium channels (Navs), voltage-gated calcium channels (VGCCs), and voltage-gated potassium channels (Kvs) at its *trans*-state upon light at 500 nm or in the dark. Conversely, irradiation with 380 nm light turns QAQ to its *cis*-state, thereby releasing these channels from the blockade. (**d**) Optovin photo-modulates transient receptor potential ankyrin 1 (TRPA1) channel activity by two distinct mechanisms, in which, after illumination with 405 nm light, either activates TRPA1 channels directly by its conversion into a reactive form and subsequent interaction with TRPA1 cysteine residues or indirectly through the generation of singlet oxygen from the surrounding aqueous oxygen. Panel (**c**) was adapted from Mourot et al., 2013 [117].

Diverse opto-pharmacological agents have been developed on the basis of photo-modulating TRP channel activity in order to photo-control nociception, [33,39,110,111,112]. In this manner, TRPV1 activity was first photo-activated by caged capsaicin compounds, which activate TRPV1 under irradiation with light [113,114,115,116] (Figure 2a), but in spite of having a substantial increase in the level of light-controlled TRPV1 activity, the caged capsaicin effect is not reversible, thus limiting the possibility to photo-control TRPV1 activity by repeated activation/deactivation cycles.

This limitation was later overcome by Trauner’s lab with the development of reversible photo-switchable TRPV1 antagonists by the inclusion of the azobenzene moiety into the structure of the TRPV1 antagonists capsazepine and BCTC, which enable a fully reversible photo-blocking of TRPV1 activity in addition to an orchestrated use in conjunction with a TRPV1 agonist [110]. Among a set of photo-switchable capsazepine derivatives, Azo-capsazepine 4 (AC4) has proven to be the most effective antagonist of TRPV1. In HEK cells heterologously expressing TRPV1, AC4 acts primarily as a *trans* antagonist (light at 440 nm) over voltage-evoked TRPV1 currents, or as a *cis* antagonist (360 nm) on TRPV1 currents activated by capsaicin. The BCTC derivative ABCTC functions preferentially as a *cis* antagonist (370 nm) on voltage-evoked TRPV1 currents, while it does not display any light-dependent effect on TRPV1 currents activated by capsaicin. 

Later, the same group designed the first azobenzene-based, reversible, photo-switchable TRPV1 agonist by generating a number of photo-lipids whose structure is comprised of the capsaicin head group and an azobenzene-containing photo-switchable fatty acid tail (AzCAs). Among them, Azo-capsaicin 4 (AzCA4) turned out to be the most effective compound to photoactivate TRPV1 channels [111]. Upon ultraviolet illumination, AzCA4 photo-stimulates natively expressed TRPV1 channels (Figure 2b) on cultured mouse DRG neurons, and these responses are, in turn, sensitized by the endogenous inflammatory agents, bradykinin and serotonin. Notably, AzCA4 can be used in combination with an optical recording tool, as demonstrated by the measurement of AzCA4 optically evoked intracellular Ca^2+^ increases in isolated TRPV1-expressing DRG neurons from a genetically modified mouse line encoding the calcium indicator GCaMP3. Ultimately, in a more physiological approach, AzCA4 was shown to photoactivate TRPV1-expressing C-fiber nociceptors from the saphenous nerve in an ex vivo skin-nerve preparation [111].

The first opto-pharmacological agent proven to photo-modulate mammalian nociception in vivo was quaternary ammonium-azobenzene-quaternary ammonium (QAQ), a photo-isomerizable molecule developed by Kramer’s lab, based on the lidocaine derivative QX-314 [118,119,120]. More recently, Trauner’s lab generated PhotoS1P, an azobenzene-based photo-switchable analog of Sphingosine-1-phosphate, which has the ability of optically controlling skin nociception in vivo at the mouse paw, by alternating 365/460 nm light irradiation [121]. Unlike the aforementioned azobenzene-containing compounds, neither QAQ nor PhotoS1P are able to directly photo-modulate TRPV1 activity, however, interestingly, QAQ is membrane-impermeant and selectively accesses nociceptive neurons through endogenous TRPV1 channels due to their pore-dilation capacity after exposure to its agonist capsaicin [122]. In this manner, QAQ intracellularly accumulates in TRPV1-expressing neurons and subsequently photo-sensitizes them by blocking voltage-gated channels in the *trans* configuration either in dark conditions or upon illumination with 500 nm light (Figure 2c). Conversely, this photo-blockade can be rapidly reverted upon irradiation of light at 380 nm. In line with this, QAQ selectively enters HEK cells transiently expressing TRPV1 channels upon activation with capsaicin, but not cells expressing TRPM8 or TRPA1 channels after being activated by menthol or allyl isothiocyanate, respectively. Moreover, QAQ photo-sensitizes nociceptor activity at three different parts of nociceptive neurons, that is, their cell bodies in the DRG, their central terminals in the spinal cord, and their peripheral sensory nerve endings. With respect to the latter case, in rat corneas treated with capsaicin, topical application of QAQ increases the mechanical threshold in response to Von Frey’s hair stimulation. This decreased mechanical sensitivity was rapidly reverted upon irradiation with light at 380 nm. This experiment demonstrates, for the first time, in vivo opto-pharmacological regulation of pain [112].

In vivo photo-control of corneal nociceptor activity takes advantage of unique corneal features such as transparency and free nerve endings located a few micrometers below the surface, thus facilitating optical control. However, the short wavelength of light needed for photo-isomerization of QAQ from and PhotoS1P *trans* to *cis* precludes their more extended use in vivo due to potential cell damage and poor penetration into intact tissues. This technical limitation might be overcome with the recent design by Kramer’s lab of a red-shifted version of QAQ (QENAQ) that photo-isomerizes to *cis*-state upon irradiation with blue light and relaxes back to *trans* in the dark in a matter of seconds [123]. In more general terms, the development of red-shifted optical tools might expand their use as photo-modulators of nociceptor activity in vivo as well as potential therapeutic agents [124,125].

Opto-pharmacological regulation of the TRPA1 channel was first achieved by means of optovin, a rhodamine-containing small molecule that, upon violet light exposure, enables reversible optical control of neural activity in zebrafish and mice [126]. Optovin was found by Peterson’s lab in a behavior-based chemical screening searching for compounds that could drive light-dependent motor behaviors in the wild-type zebrafish. Specifically, optovin enables in vivo photo-control of zebrafish dorsal fin movements in a TRPA1-dependent manner and additionally has been shown to photoactivate human TRPA1 channels heterologously expressed in HEK293 cells and the mouse TRPA1 channels natively expressed in cultured sensory DRG neurons. Experimental data on the mechanism of action points to the existence of both indirect and direct mechanisms for the photo-activation of TRPA1 channels by optovin. On the one hand, as a result of the exposition of optovin to 405 nm light, the ROS singlet oxygen is generated from the surrounding aqueous oxygen, which subsequently activates TRPA1 channels (Figure 2d). On the other hand, light irradiation induces the conversion of optovin into a reactive state compound which may directly interact with redox-sensitive cysteine residues in the TRPA1 channel through the formation of a reversible covalent thioether bond. Importantly, optovin lacks light-inducible reversibility since activation is spontaneously reversed in darkness by a currently unknown mechanism, which makes this approach somewhat similar to photo-uncaging, where the caged ligand is released upon light stimulation and deactivation requires spontaneous and often slow dissipation of the ligand, limiting their use [127]. Very recently, the same group identified two photo-isomerizable compounds, the azobenzene-containing TRPswitch-A, and the azopyrazole-containg TRPswitch-B, both bestowed with the ability to reversibly and repeatedly photo-activate the zebrafish TRPA1b. An additional advantage over optovin is that TRPswitches offer faster activation/deactivation kinetics upon illumination at UV/green light, respectively. It should be noted that despite not activating mammalian TRPA1 channels, TRPswitches are the first photo-isomerizable pharmacological tools in photo-modulating TRPA1 channels, and modification of the TRPswitch scaffold might provide ligands with different species specificity, thus expanding the nociceptive TRP channel’s opto-pharmacological toolkit [127] (Table 1). 

To date, only a few opto-pharmacological agents have been directly assessed at the nerve terminal [111,118]. Nevertheless, the outstanding progress of opto-pharmacology, has provided a significant repertoire of high flexibility and versatility light-tunable ligands and ion receptors, with photochromic tethered ligands furnishing the highest molecular specificity and photochromic ligands having the major advantage of not requiring genetic modifications of the target protein. Altogether, these growing opto-pharmacological advances bring new opportunities to tackle fundamental questions on TRP channels’ role in nociceptive nerve terminal physiology and pathology.

**Table 1:** Summarizing the opto-pharmacological compounds used for modulation of nociceptive TRP channel activity and/or nociceptive neuron activity.

Opto-Chemical Agent	Target	Activity	Opto-Chemical Strategy	Testing Method
Caged-capsaicin [116]	TRPV1	agonist	Caged ligand	in vitro: rat DRG neurons.
AC4 ^1^ [110]	TRPV1	antagonist	Azobenzene-based photochromic ligand	in vitro: recombinant rat TRPV1.
ABCTC ^2^ [110]	TRPV1	antagonist	Azobenzene-based photochromic ligand	in vitro: recombinant rat TRPV1.
AzCA4 ^3^ [111]	TRPV1	agonist	Azobenzene-based photochromic ligand	in vitro: mouse DRG neurons. ex vivo: skin nerve preparation.
Optovin [126]	TRPA1	agonist	Rhodamine-containing photochromic ligand	in vitro: recombinant human TRPA1 and mouse DRG neurons.
TRP-switch-A [127]	zTRPA1b ^4^	agonist	Azobenzene-based photochromic ligand	in vitro: recombinant zebrafish TRPA1b.
TRP-switch-B [127]	zTRPA1b ^4^	agonist	Azopyrazole-containing photochromic ligand	in vitro: recombinant zebrafish TRPA1b.
QAQ ^5^ [118]	Navs, Kvs and VGCC	antagonist	Azobenzene-based photochromic ligand	in vitro: rat TG neurons. ex vivo: mouse DRG. in vivo: Von Frey assay.
QENAQ ^6^ [123]	Navs and Kvs	antagonist	Azobenzene-based photochromic ligand	in vitro: mouse TG neurons. ex vivo: mouse DRG.
PhotoS1P [121]	S1P ^7^	agonist	Azobenzene-based photochromic ligand	in vitro: mouse DRG neurons. in vivo: Hargreaves test.

Please refer to References [41] and [128] for the reviews summarizing the classical nociceptive TRP channel modulators. ^1^ Azo-capsazepine 4. ^2^ Azo-BCTC. ^3^ Azo-capsaicin 4. ^4^ Zebrafish TRPA1b. ^5^ Quaternary ammonium-azobenzene-quaternary ammonium. ^6^ Quaternary ammonium-ethylamine-azobenzene-quaternary ammonium. ^7^ Sphingosine-1-phosphate receptors.

## 4. Optical Recording of Nociceptive TRP Channel Activity at the Nerve Terminal

Examining TRP-mediated currents using an electrophysiological approach allows low throughput but high-resolution access to the biophysics and physiology of TRP channels. The utilization of optical imaging to study Ca^2+^ or Na^+^ influx through TRP channels is beneficial for moderate-to-high throughput access to TRP function and pharmacology. However, in some cases, the optical recording approach is a necessity. One of these instances is in the analysis of structures that are inaccessible by standard electrophysiological techniques. A prominent example of this is in the study of the physiology of peripheral terminals of nociceptive neurons.

Despite being key structures in nociception, not much is known regarding the functional properties of the peripheral terminals of primary nociceptive neurons. The peripheral branches of sensory nerves are generally embedded in their surrounding tissues, thus, making a direct electrophysiological recording of the membrane potential changes, caused by natural stimulation of the thin terminal endings, difficult. This limitation was solved in the mid-20th century, using invertebrate preparations such as the stretch receptor of the crayfish [129], formed by an isolated peripheral sensory neuron whose short, brush-like dendritic branches terminate onto abdominal muscle fibers. A microelectrode inserted into the cell soma allowed to record events occurring at the nearby terminal. Similar studies were performed on the Pacinian corpuscle, which contains a single myelinated sensory nerve axon and loses its myelin and Schwann sheaths when entering the corpuscle. Application of an electrode there enabled the simultaneous extracellular recording of the electrotonic extension along the axon of the membrane potential change evoked by weak mechanical force applied to the corpuscle’s surface (the generator potential) and of the propagated action potentials generated by the receptor potential at the first node of Ranvier [130]. Altogether, these pioneering studies defined the general characteristics of the process of excitation of the peripheral endings of mammalian primary somatosensory neurons. However, the weak currents generated at the minute nerve terminals of the thermal and nociceptive unmyelinated primary sensory neurons could not be directly recorded.

Impulse firing in single axons of nociceptive neurons, which codes specific stimuli, have been directly analyzed in platforms such as the skin-nerve preparation and the ex-vivo somatosensory system model [131,132]. These allow for a direct assessment of activity patterns of fibers or terminals, but not the biophysical mechanism underlying this activity. Electrical recordings of the cell body or of membrane patches of primary sensory neurons of different modalities have also been used to further define the contribution of TRP and voltage-gated channels to the generator potential and specific responsiveness to their natural stimuli. However, considering that the terminal’s functional environment [133,134,135,136,137], geometry [25,26,138], and likely its passive membrane properties [19,139] differ from cell bodies situated in DRGs or TGs, the actual detection and transmission of noxious stimuli may be significantly different from predictions made based on data obtained from studying neuronal somata. Thus, some of the basic questions in nociception and pain cannot be answered by solely studying cell bodies. For example, studies from DRG can predict, but not directly answer, what molecular composites are involved in terminal generator potential. Also, studying only DRG somata makes it impossible to determine the contribution of TRP and voltage-gated channels to the generator potential and the location of the transition between the generator potential to action potential. Moreover, a recent computational study predicted that nociceptive output resulting from activation of terminal TRPV1 channels depends on the terminal tree structure [19], emphasizing once again the importance of direct investigation of signal propagation along nociceptive terminals. Our knowledge of normal and abnormal transmission of pain signals is dependent on having a full understanding of the occurrences in the nerve terminals that lead to the sensation of pain. New methods for extracellular recordings from single nociceptor nerve terminals in guinea-pig corneas [140,141] and whole-cell recordings from the terminal bulb in cultured DRG neurons [142], contribute essential information, showing that free nerve endings of mammalian primary sensory neurons subserving separate stimulus modalities possess different membrane characteristics associated with the propagation of nerve impulses in their peripheral nerve terminals, and provide physiologically relevant information regarding the role of specific channels in nociceptive terminal activity.

Ion imaging of terminals, however, adds several advantages to electrophysiological recordings. Firstly, ion imaging provides sufficient spatial and temporal resolution to study the ion dynamics of detection and propagation of noxious stimuli, in detail, along the terminal and distal axon. Additionally, electrophysiological recordings involve applying the recording electrodes that could injure the tissue and thus lead, in itself, to the activation of nociceptive terminals. Optical imaging does not involve electrode insertion and, therefore, could be performed in intact tissue. Lastly, imaging from terminals could be performed ex vivo and in vivo from multiple terminals simultaneously. The disadvantage of this approach is that there may be insufficient time resolution to characterize fast time-dependent kinetics of ion channels. This disadvantage was addressed recently using an ultrasensitive imaging apparatus, allowing sampling of GCaMP6-emitted Ca^2+^ signals in a compartmental chamber from cultured terminal-like structures of nociceptive neurons at 2 kHz [25]. Such an approach allowed for studying signal generation and propagation in a millisecond time resolution and 1 µm space resolution. In this model, the focal calibrated application of capsaicin onto a single terminal evoked distinctive Ca^2+^ signals along the nociceptive terminal-like process. Using this near-action potential time resolution, the authors demonstrated that application of capsaicin in the presence of Nav blockers leads to an increase in TRPV1-mediated intra-terminal Ca^2+^, which slowly decays as it propagates along the nociceptive fiber [25]. This approach was sensitive enough to detect differences in latency, time-to-peak, and amplitude of capsaicin-induced ion transients along the terminal neurites. Importantly, this approach allowed, for the first time, to study TRPV1-mediated Na^+^ signals at the nociceptive terminal-like processes, using a Na^+^-sensitive dye, sodium-binding benzofuran isophthalate (SBFI). From a methodological point of view, this in vitro approach, which is based on a distinctive compartment containing only neurite terminals, is currently the only approach that allows the direct study of terminals’ Nav dynamics. In this regard, it is even advantageous over an in vivo assay as the usage of acetoxymethyl (AM)-based dyes in vivo will likely produce strong background noise due to AM dye loading of non-neuronal cells in the imaged area. Nonetheless, the significant limitation of this approach is that the recordings were performed in vitro from the terminal-like processes, whose growth was induced from the cultured DRG neurons by applying various concentrations of NGF. Thus, this model does not necessarily represent the terminal behavior in its natural conditions. Importantly, the properties of capsaicin-induced signal propagation in these cultured terminal-like structures were similar to the propagation of capsaicin-induced signals recorded from corneal nociceptive terminals recorded in vivo in intact tissue [26], which will be discussed in detail below.

The hurdle in studying the nociceptive terminals in a natural environment using optical recordings, in physiologically relevant time resolution, is that layers of non-transparent tissue, like skin, optically obscure most of the peripheral sensory nerve terminals. Imaging of nociceptive terminal activity in paw skin in vivo or ex vivo requires confocal or multiphoton microscopy with long (seconds) frame acquisition times. Recently, Ca^2+^ signals from terminal branches of skin nociceptive neurons ex vivo were analyzed using a spinning disk confocal microscope at a 0.25 Hz acquisition rate [27]. The authors recorded the activity of nociceptive terminal branches in a skin-nerve preparation consisting of a skin flap from the hind paw’s dorsal surface and the innervating saphenous nerve from mice expressing GCaMP3 in TRPV1-expressing neurons. The skin from the dorsal surface was utilized since the plantar skin tissue is significantly thicker and less amenable for imaging. Due to the low drug permeability of the epidermis, the skin flap was placed with the dermis side up, and TRP channel agonists were applied from above, thus the imaging of the terminals was performed from the epidermal side using an inverted confocal microscope. Imaging of the skin nociceptive terminal branches while applying various activators of TRP channels revealed increased activity of TRPM3 channels during complete Freund’s adjuvant (CFA)-mediated skin inflammation, emphasizing the role of terminals’ TRPM3 channels in inflammatory pain [27].

Interestingly, some skin areas, such as from ear explants, allow faster (2.6 Hz) single-photon confocal imaging of nociceptive terminal activity [28]. Using Ca^2+^ imaging from nociceptive fibers expressing GCaMP3 from acutely isolated hairy ear skin explants, Kim and colleagues were able to assess TRPV1-induced activity of multiple peripheral nociceptive terminals’ fibers and reveal that the terminals of uninjured trigeminal V3 nerves show extensive TRPV1 hyperactivity following nerve injury to the trigeminal V2 branch [28].

To record the activity of nociceptive terminals innervating the target organs, some investigators take advantage of easily accessible and transparent tissues such as cornea and dura mater, which are abundantly innervated by nociceptive neurons [143,144]. Mulligan’s lab recently introduced an ex vivo preparation containing a skull bone with arachnoid and dura mater attached. To assess the activity of nociceptive fibers innervating dura mater in this preparation, membrane-permeant Ca^2+^ indicator Rhod-2 AM was applied directly on the preparation, and nociceptive terminal branches were visualized using wide-field epifluorescence microscopy [29]. The activity of TRPV1 channels in these terminal branches was accessed using UV-photolysis of caged capsaicin, as described above (Figure 2a), and the resulting activity was recorded as it propagated along the fiber [29]. This approach was utilized to record the activity of dural CGRP-expressing terminal fibers and show the effects of 5-HT1 receptors and µ-opioid receptors on the electrically evoked activity of nociceptive terminal fibers [145,146].

Corneal tissue, which is primarily innervated by nociceptive neurons [147,148] (see also above) and is easily accessible for manipulations, has been widely used for the electrical recordings of nociceptive terminals [140,141] and recently for ex vivo [30,149] and in vivo [26] optical recordings from nociceptive terminals.

To study the molecular basis of TRPV1-mediated terminal activity, Weinreich’s lab performed fluorescent measurements of capsaicin-evoked Ca^2+^ signals ex vivo from isolated excised corneas or corneas in isolated eyes. Ca^2+^ signals were tagged by soaking the rat corneal tissue or whole eye in a Ca^2+^ indicator, Oregon Green 488 BAPTA-1 [149]. In later experiments, Oregon Green 488 BAPTA or another Ca^2+^ indicator FURA-2 dextran were applied in vivo, and corneas were then excised for imaging [30]. Ca^2+^ imaging (at a 0.5 to 1.7 Hz acquisition rate) was obtained using epifluorescence microscopy following focal application of capsaicin or electrical stimulation [149]. Treatment with capsaicin triggered Ca^2+^ signals at the terminal fibers, which were fully blocked by applying TRPV1 channel antagonist, capsazepine [149]. Importantly, these signals were not affected by blocking VGCCs [149].

**Figure 3:**
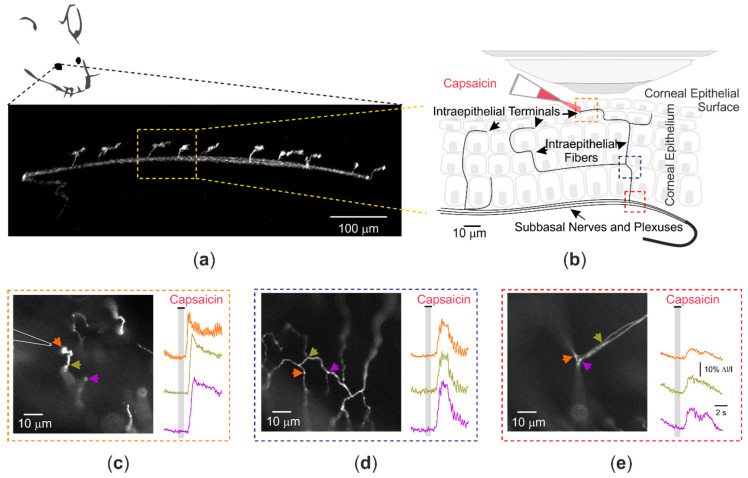
In vivo optical recording from nociceptive corneal nerve terminals and distal axons showing initiation and propagation of capsaicin-induced Ca^2+^ signals. (**a**) Transversal fluorescence reconstruction of a sub-basal nerve, giving rise to several terminals within the corneal epithelium cells. (**b**) Scheme of the experimental preparation in which a non-invasive calibrated puff of capsaicin was used to stimulate corneal nociceptive nerve terminals. The capsaicin-evoked Ca^2+^ transients were recorded along (**c**) the nociceptive terminal tips, (**d**) the terminal fibers, and (**e**) deeper into their parental plexuses. The corresponding areas in the transversal plane are shown in (**b**) (color-coded boxes). The traces (right panels) depict the changes in GCaMP6s fluorescence following application of capsaicin onto the terminal tip and they are color-coded according to the corresponding regions of interest (arrows shown in left panels). Reproduced from Goldstein et al., 2019 [26], with permission.

Although in vitro and ex vivo recordings already revealed important information regarding the activity of peripheral nociceptive terminals in general and TRPV1 channels specifically, to characterize these processes in the most physiological conditions is crucial to be able to test these same premises in vivo. While basing the development of the methodologies employed above, a novel technique was developed to study nociceptive terminals, specifically TRPV1 channels, in an in vivo framework [26] (Figure 3a).

In this approach, the established benefit of corneal peripheral nociceptive terminals was utilized, and mice’s corneal nociceptive neurons were infected with Adeno-Associated Virus serotype-1, carrying an expression cassette for GCaMP6s. Ten to fourteen days after the virus injection, the recordings were performed from nociceptive terminals and terminal fibers innervating corneas in anesthetized mice. Similarly to the in vitro experiment [25], a focal calibrated puff was used to apply capsaicin onto an individual terminal, activating the terminal’s TRPV1 channels (Figure 3b). Notably, the puff pipette was placed just above the corneal epithelium such that it would not affect tissue integrity. Thus, these experiments have the advantage of the capacity to record the activity of individual terminals following the application of “natural” noxious stimuli in the physiological condition in vivo within intact tissue. The detection was performed using an ultrasensitive imaging apparatus used previously to detect changes in intra-axonal Na^+^ concentrations in central neurons with time resolution of single action potential [150], and to record capsaicin-mediated changes in Ca^2+^ and Na^+^ signals in cultured terminal-like processes, as explained above [25]. The ultrasensitive apparatus, together with optical properties of corneal tissue, allowed for acquiring changes in GCaMP6s fluorescence in vivo with a relatively high frame rate of 40–125 Hz. It also permitted the recording of changes of Ca^2+^ following focal terminal stimulation, not just at the terminal tip (Figure 3c) but also deeper at the level of terminal fibers (15–30 µm depth from the corneal surface) (Figure 3d) and even plexuses (60–100 µm depth from the corneal surface) [26] (Figure 3e). This approach allowed to measure the capsaicin-mediated activity of terminal TRPV1 channels in vivo in intact tissue. It demonstrated that the terminal capsaicin-evoked Ca^2+^ signals were not sensitive to the application of Na^+^ channel blockers, however, the application of benedipine, a T-, N-, and L-type VGCC blocker, reduced the signal amplitude. These data show that in mice nociceptive neurons in vivo, TRPV1-mediated signal is amplified by the activation of VGCCs [26]. The recordings of TRPV1-induced activity in vivo also revealed that the TRPV1- and VGCC-mediated depolarization propagates passively along the terminal for about 25–30 µm, and only then do Navs contribute to the signal, defining the location of the spike initiation zone at the nociceptive terminals. Using this approach, authors were also able to study the changes in TRPV1-induced signal generation and propagation in mice with acute corneal inflammation, demonstrating that in such instances, the TRPV1-mediated signal at the terminal tip is also amplified by the activation of Navs at the terminal tip, which become functionally available under inflammatory conditions. This additional amplification of the TRPV1-induced signal caused an increase in the excitability of nociceptive neurons, leading to inflammatory pain.

In summary, various in vitro, ex vivo, and in vivo approaches for the optical recordings of the activity of peripheral nociceptive terminal opens unique windows into understanding the activity of these tiny structures, which defines pain sensation in normal and pathological conditions.

## 5. Concluding Remarks

In the last two decades, our understanding of the molecular mechanisms underlying nociception has advanced in an unparalleled manner since the identification and cloning of nociceptive receptors, mainly belonging to the TRP channel superfamily. Undoubtedly, the extensive use of in vitro and ex vivo electrophysiological and ion imaging recordings, simultaneously with a myriad of pharmacological compounds and genetically engineered mice targeting nociceptive TRP channels, has provided invaluable information on the role that TRP channels play in the primary nociceptive neuron.

Nevertheless, the limited electrophysiological accessibility to peripheral nerve terminals, along with either invasive or low spatiotemporal precision methods to control endogenous ion channel activity in their physiological context, have hindered a direct assessment of TRP channel function at the nociceptive nerve terminal. Recent advances in the development of optical tools and methods for both recording and control of nociceptive TRP channel activity open avenues for uncovering pivotal aspects of the biology of nociception and pave the way for new applications in precision medicine of pain.
